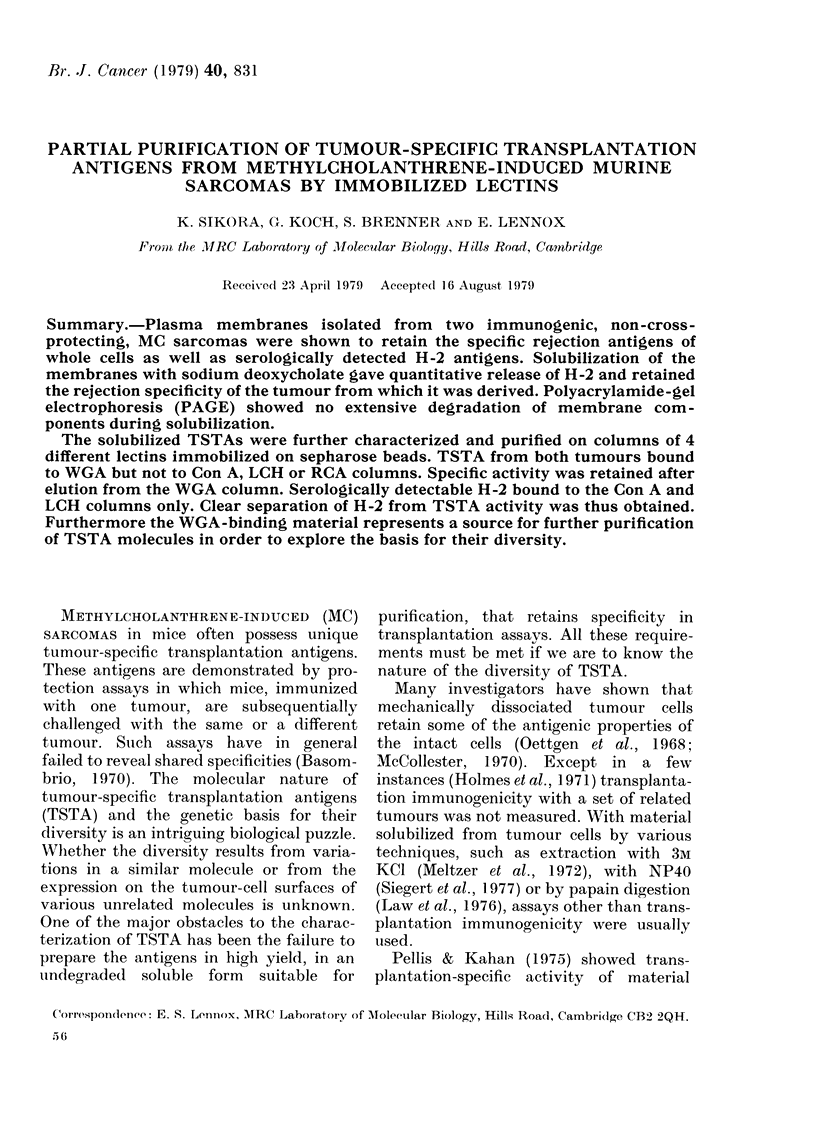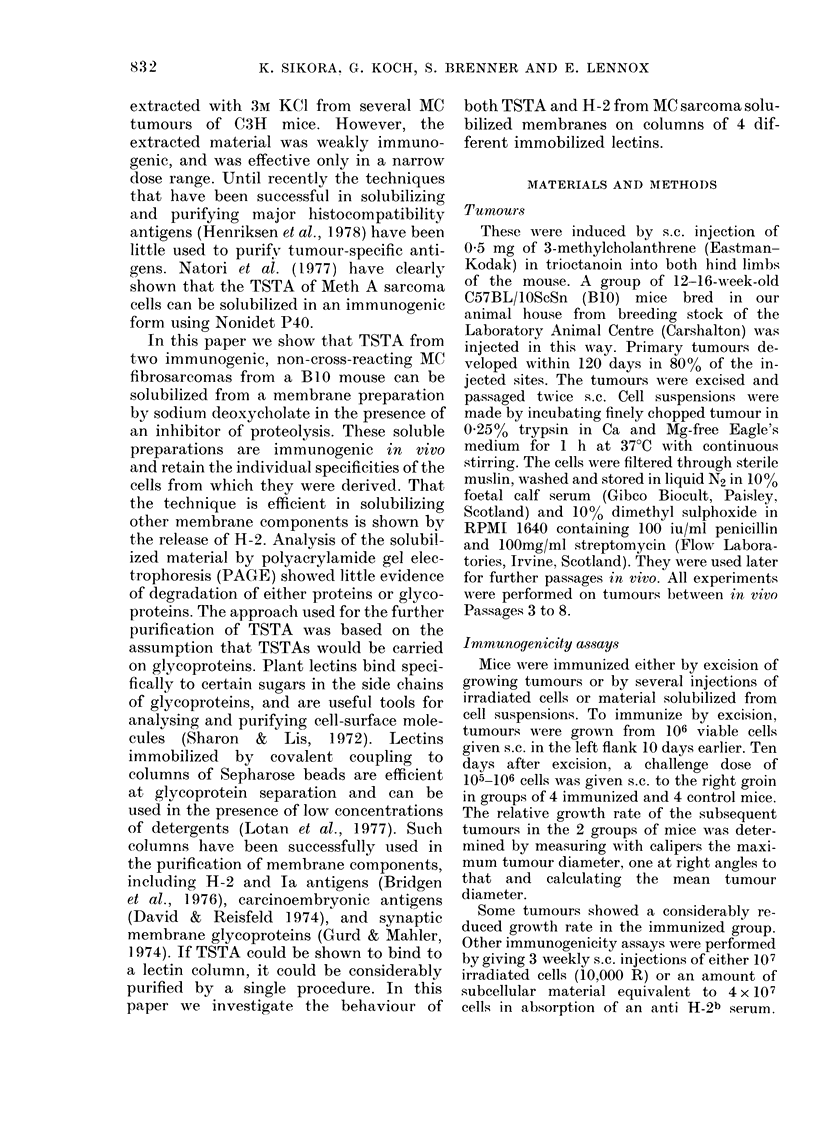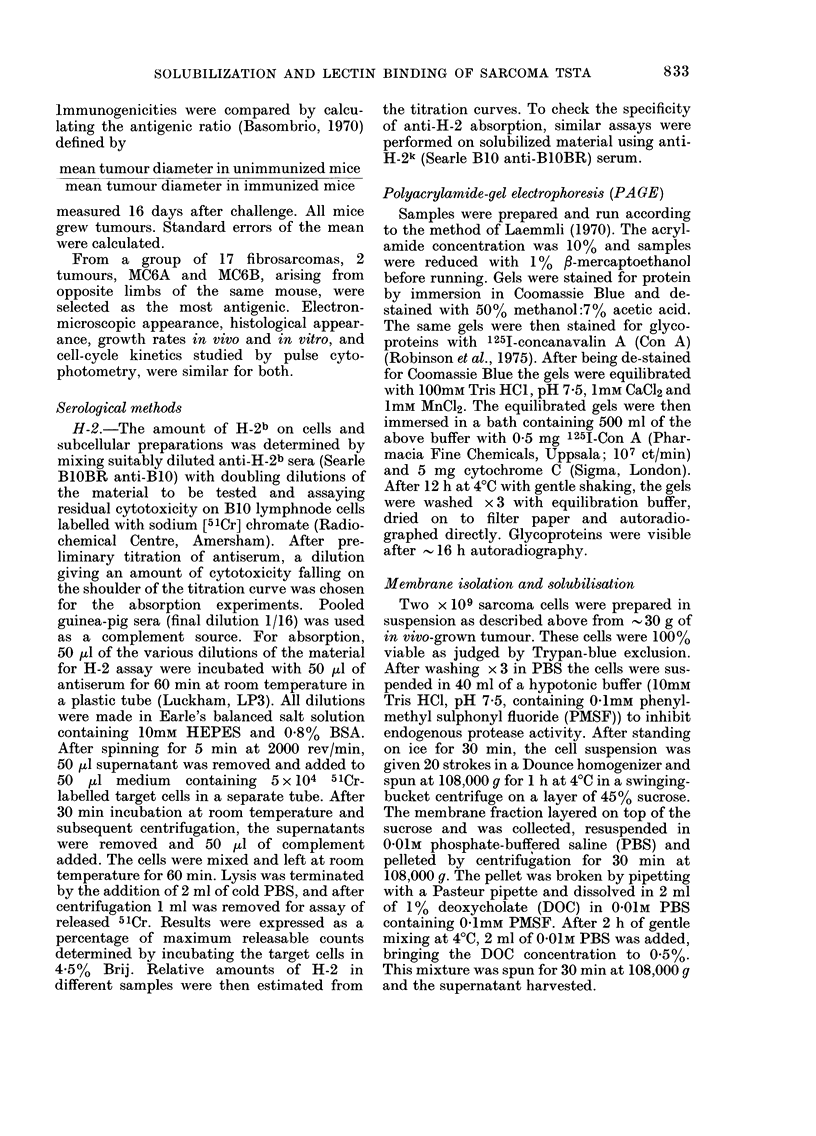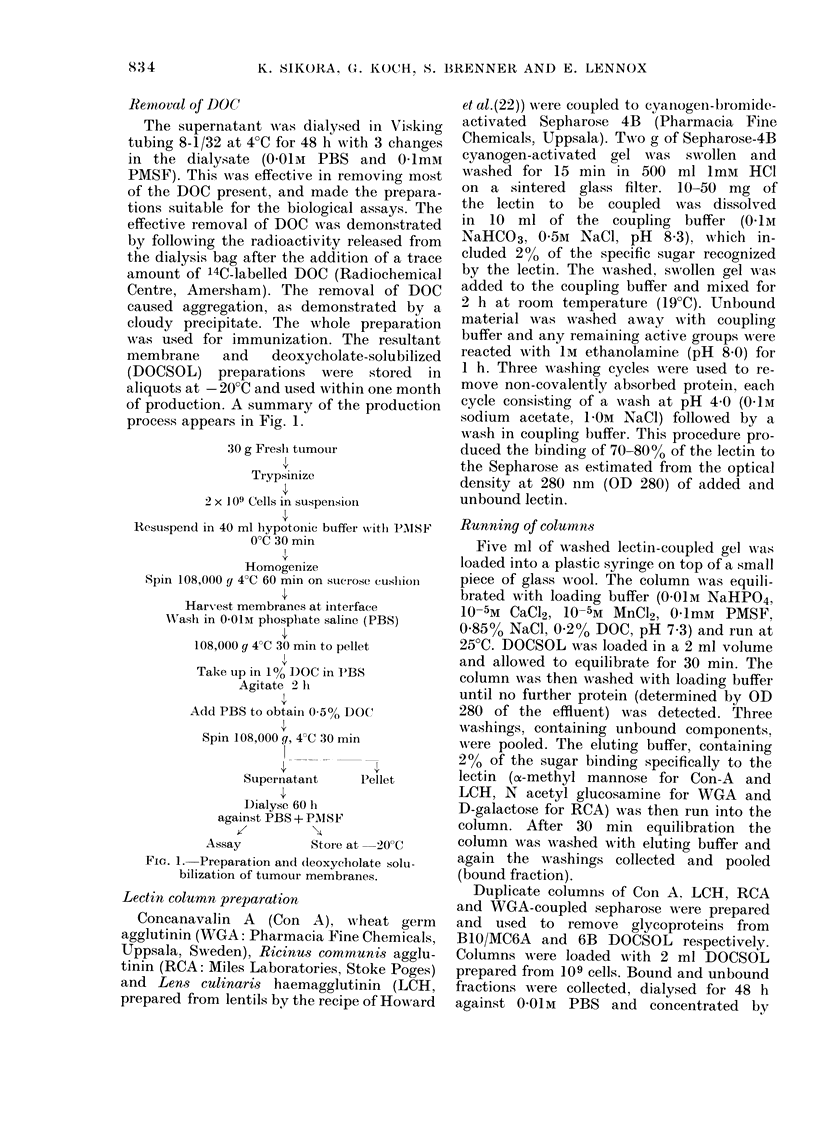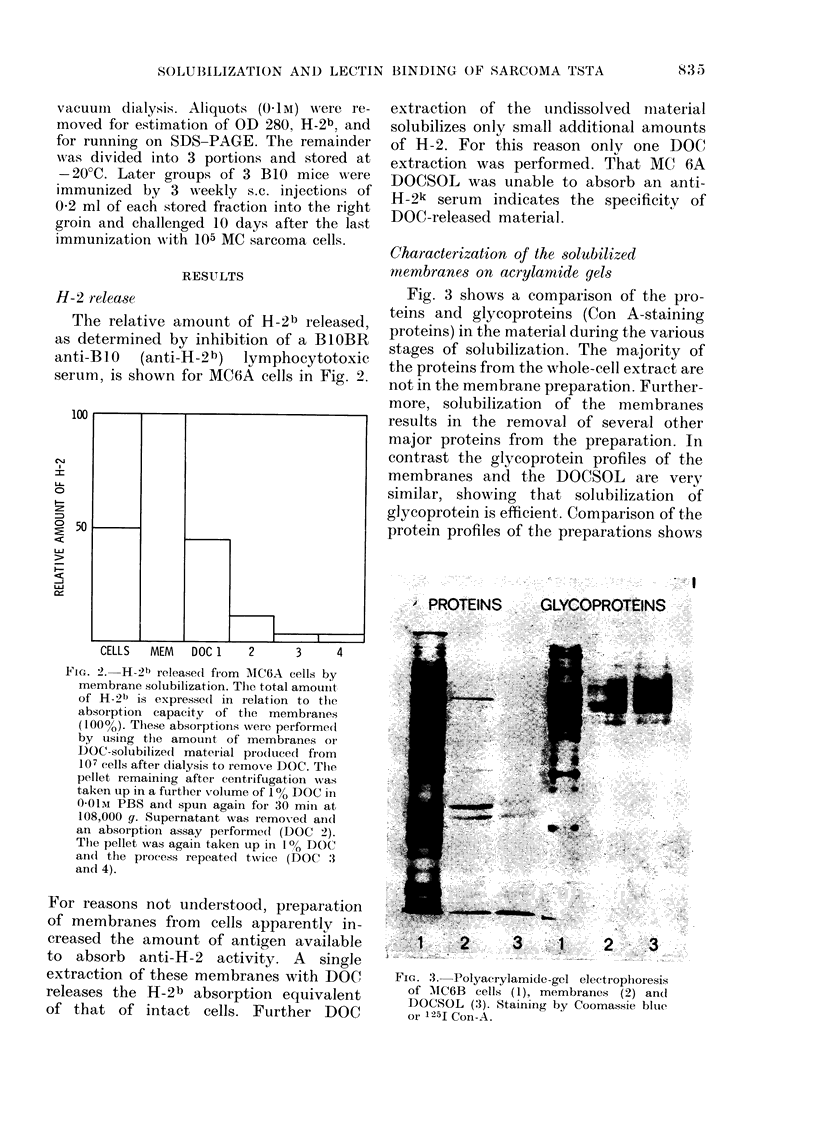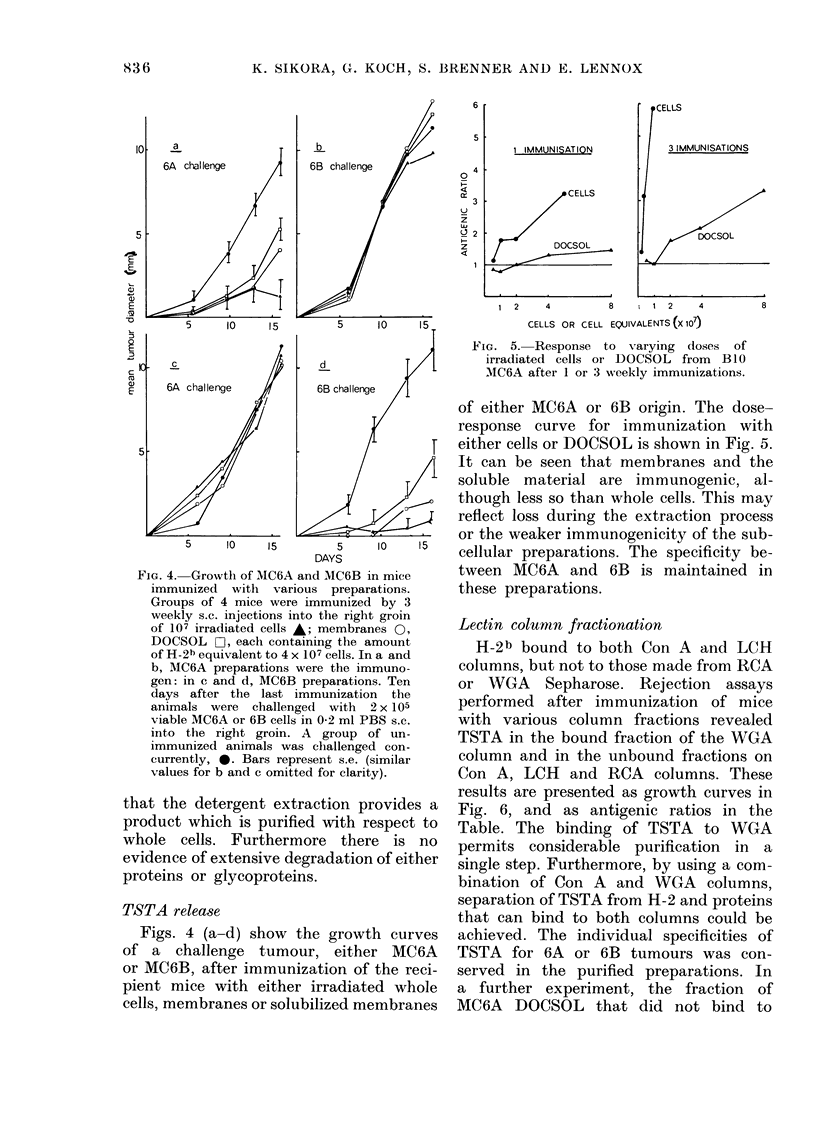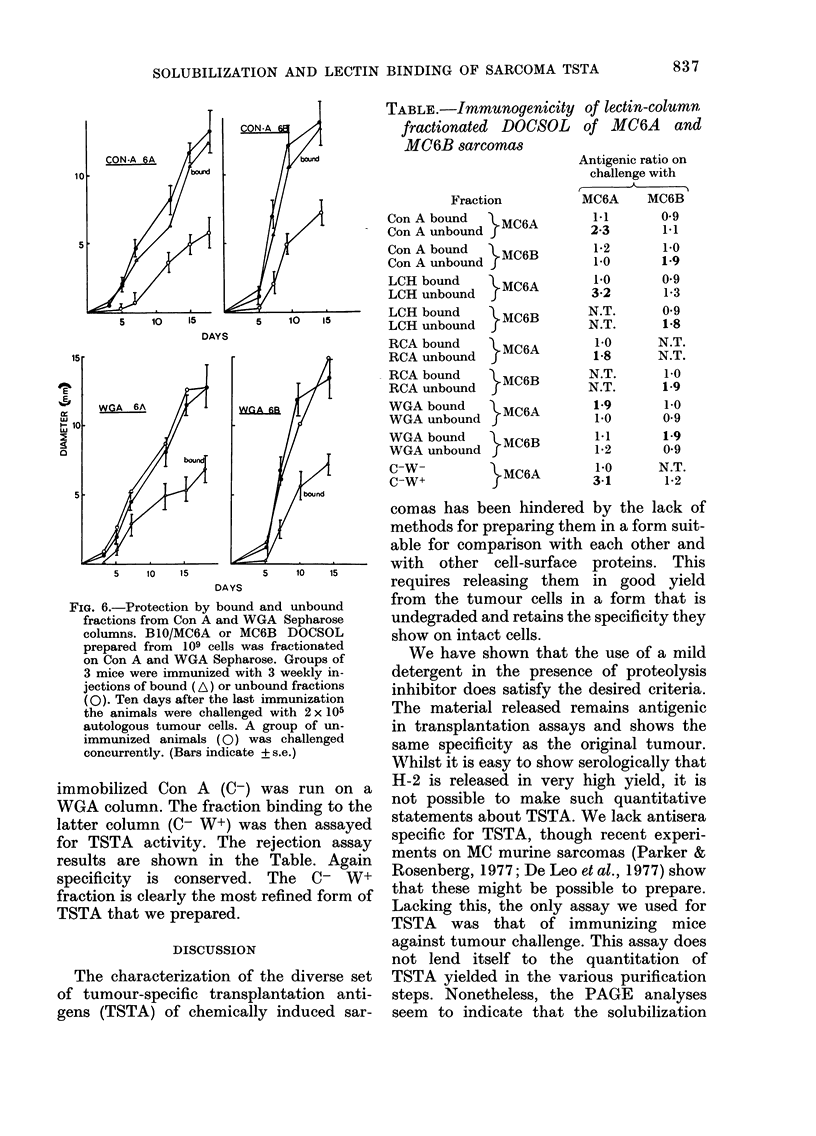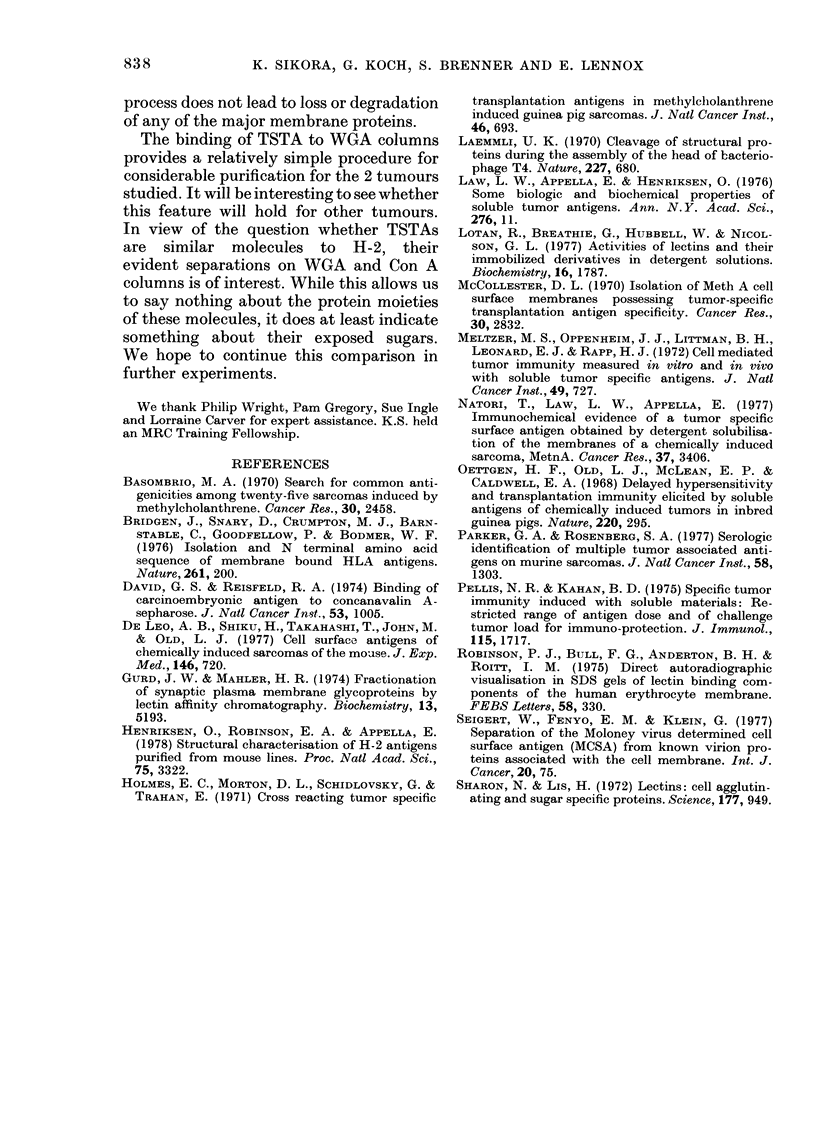# Partial purification of tumour-specific transplantation antigens from methylcholanthrene-induced murine sarcomas by immobilized lectins.

**DOI:** 10.1038/bjc.1979.273

**Published:** 1979-12

**Authors:** K. Sikora, G. Koch, S. Brenner, E. Lennox

## Abstract

**Images:**


*Br. J. Cancer* (1979) **40**, 831

### PARTIAL PURIFICATION OF TUMOUR-SPECIFIC TRANSPLANTATION ANTIGENS FROM METHYLCHOLANTHRENE-INDUCED MURINE SARCOMAS BY IMMOBILIZED LECTINS

K. SIKORA, G. KOCH, S. BRENNER AND E. LENNOX

*From the MRC Laboratory of Molecular Biology, Hills Road, Cambridge*

Received 23 April 1979 Accepted 16 August 1979

**Summary.—Plasma membranes isolated from two immunogenic, non-cross-protecting, MC sarcomas were shown to retain the specific rejection antigens of whole cells as well as serologically detected H-2 antigens. Solubilization of the membranes with sodium deoxycholate gave quantitative release of H-2 and retained the rejection specificity of the tumour from which it was derived. Polyacrylamide-gel electrophoresis (PAGE) showed no extensive degradation of membrane components during solubilization.**

**The solubilized TSTAs were further characterized and purified on columns of 4 different lectins immobilized on sepharose beads. TSTA from both tumours bound to WGA but not to Con A, LCH or RCA columns. Specific activity was retained after elution from the WGA column. Serologically detectable H-2 bound to the Con A and LCH columns only. Clear separation of H-2 from TSTA activity was thus obtained. Furthermore the WGA-binding material represents a source for further purification of TSTA molecules in order to explore the basis for their diversity.**

METHYLCHOLANTHRENE-INDUCED (MC) SARCOMAS in mice often possess unique tumour-specific transplantation antigens. These antigens are demonstrated by protection assays in which mice, immunized with one tumour, are subsequentially challenged with the same or a different tumour. Such assays have in general failed to reveal shared specificities (Basombrio, 1970). The molecular nature of tumour-specific transplantation antigens (TSTA) and the genetic basis for their diversity is an intriguing biological puzzle. Whether the diversity results from variations in a similar molecule or from the expression on the tumour-cell surfaces of various unrelated molecules is unknown. One of the major obstacles to the characterization of TSTA has been the failure to prepare the antigens in high yield, in an undegraded soluble form suitable for purification, that retains specificity in transplantation assays. All these requirements must be met if we are to know the nature of the diversity of TSTA.

Many investigators have shown that mechanically dissociated tumour cells retain some of the antigenic properties of the intact cells (Oettgen *et al.*, 1968; McCollester, 1970). Except in a few instances (Holmes *et al.*, 1971) transplantation immunogenicity with a set of related tumours was not measured. With material solubilized from tumour cells by various techniques, such as extraction with 3M KCl (Meltzer *et al.*, 1972), with NP40 (Siegert *et al.*, 1977) or by papain digestion (Law *et al.*, 1976), assays other than transplantation immunogenicity were usually used.

Pellis & Kahan (1975) showed transplantation-specific activity of material

Correspondence: E. S. Lennox, MRC Laboratory of Molecular Biology, Hills Road, Cambridge CB2 2QH.

extracted with 3M KCl from several MC tumours of C3H mice. However, the extracted material was weakly immunogenic, and was effective only in a narrow dose range. Until recently the techniques that have been successful in solubilizing and purifying major histocompatibility antigens (Henriksen *et al.*, 1978) have been little used to purify tumour-specific antigens. Natori *et al.* (1977) have clearly shown that the TSTA of Meth A sarcoma cells can be solubilized in an immunogenic form using Nonidet P40.

In this paper we show that TSTA from two immunogenic, non-cross-reacting MC fibrosarcomas from a B10 mouse can be solubilized from a membrane preparation by sodium deoxycholate in the presence of an inhibitor of proteolysis. These soluble preparations are immunogenic *in vivo* and retain the individual specificities of the cells from which they were derived. That the technique is efficient in solubilizing other membrane components is shown by the release of H-2. Analysis of the solubilized material by polyacrylamide gel electrophoresis (PAGE) showed little evidence of degradation of either proteins or glycoproteins. The approach used for the further purification of TSTA was based on the assumption that TSTAs would be carried on glycoproteins. Plant lectins bind specifically to certain sugars in the side chains of glycoproteins, and are useful tools for analysing and purifying cell-surface molecules (Sharon & Lis, 1972). Lectins immobilized by covalent coupling to columns of Sepharose beads are efficient at glycoprotein separation and can be used in the presence of low concentrations of detergents (Lotan *et al.*, 1977). Such columns have been successfully used in the purification of membrane components, including H-2 and Ia antigens (Bridgen *et al.*, 1976), carcinoembryonic antigens (David & Reisfeld 1974), and synaptic membrane glycoproteins (Gurd & Mahler, 1974). If TSTA could be shown to bind to a lectin column, it could be considerably purified by a single procedure. In this paper we investigate the behaviour of both TSTA and H-2 from MC sarcoma solubilized membranes on columns of 4 different immobilized lectins.

### MATERIALS AND METHODS

### *Tumours*

These were induced by s.c. injection of 0·5 mg of 3-methylcholanthrene (Eastman-Kodak) in trioctanoin into both hind limbs of the mouse. A group of 12–16-week-old C57BL/10ScSn (B10) mice bred in our animal house from breeding stock of the Laboratory Animal Centre (Carshalton) was injected in this way. Primary tumours developed within 120 days in 80% of the injected sites. The tumours were excised and passaged twice s.c. Cell suspensions were made by incubating finely chopped tumour in 0·25% trypsin in Ca and Mg-free Eagle's medium for 1 h at 37°C with continuous stirring. The cells were filtered through sterile muslin, washed and stored in liquid $N_2$ in 10% foetal calf serum (Gibco Biocult, Paisley, Scotland) and 10% dimethyl sulphoxide in RPMI 1640 containing 100 iu/ml penicillin and 100mg/ml streptomycin (Flow Laboratories, Irvine, Scotland). They were used later for further passages *in vivo*. All experiments were performed on tumours between *in vivo* Passages 3 to 8.

### *Immunogenicity assays*

Mice were immunized either by excision of growing tumours or by several injections of irradiated cells or material solubilized from cell suspensions. To immunize by excision, tumours were grown from $10^6$ viable cells given s.c. in the left flank 10 days earlier. Ten days after excision, a challenge dose of $10^5$–$10^6$ cells was given s.c. to the right groin in groups of 4 immunized and 4 control mice. The relative growth rate of the subsequent tumours in the 2 groups of mice was determined by measuring with calipers the maximum tumour diameter, one at right angles to that and calculating the mean tumour diameter.

Some tumours showed a considerably reduced growth rate in the immunized group. Other immunogenicity assays were performed by giving 3 weekly s.c. injections of either $10^7$ irradiated cells (10,000 R) or an amount of subcellular material equivalent to $4 \times 10^7$ cells in absorption of an anti $H\text{-}2^b$ serum.

Immunogenicities were compared by calculating the antigenic ratio (Basombrio, 1970) defined by

$$\frac{\text{mean tumour diameter in unimmunized mice}}{\text{mean tumour diameter in immunized mice}}$$

measured 16 days after challenge. All mice grew tumours. Standard errors of the mean were calculated.

From a group of 17 fibrosarcomas, 2 tumours, MC6A and MC6B, arising from opposite limbs of the same mouse, were selected as the most antigenic. Electron-microscopic appearance, histological appearance, growth rates *in vivo* and *in vitro*, and cell-cycle kinetics studied by pulse cytophotometry, were similar for both.

*Serological methods*

*H-2.*—The amount of H-$2^b$ on cells and subcellular preparations was determined by mixing suitably diluted anti-H-$2^b$ sera (Searle B10BR anti-B10) with doubling dilutions of the material to be tested and assaying residual cytotoxicity on B10 lymphnode cells labelled with sodium [$^{51}$Cr] chromate (Radiochemical Centre, Amersham). After preliminary titration of antiserum, a dilution giving an amount of cytotoxicity falling on the shoulder of the titration curve was chosen for the absorption experiments. Pooled guinea-pig sera (final dilution 1/16) was used as a complement source. For absorption, 50 $\mu$l of the various dilutions of the material for H-2 assay were incubated with 50 $\mu$l of antiserum for 60 min at room temperature in a plastic tube (Luckham, LP3). All dilutions were made in Earle's balanced salt solution containing 10mM HEPES and 0·8% BSA. After spinning for 5 min at 2000 rev/min, 50 $\mu$l supernatant was removed and added to 50 $\mu$l medium containing $5 \times 10^4$ $^{51}$Cr-labelled target cells in a separate tube. After 30 min incubation at room temperature and subsequent centrifugation, the supernatants were removed and 50 $\mu$l of complement added. The cells were mixed and left at room temperature for 60 min. Lysis was terminated by the addition of 2 ml of cold PBS, and after centrifugation 1 ml was removed for assay of released $^{51}$Cr. Results were expressed as a percentage of maximum releasable counts determined by incubating the target cells in 4·5% Brij. Relative amounts of H-2 in different samples were then estimated from the titration curves. To check the specificity of anti-H-2 absorption, similar assays were performed on solubilized material using anti-H-$2^k$ (Searle B10 anti-B10BR) serum.

*Polyacrylamide-gel electrophoresis (PAGE)*

Samples were prepared and run according to the method of Laemmli (1970). The acrylamide concentration was 10% and samples were reduced with 1% $\beta$-mercaptoethanol before running. Gels were stained for protein by immersion in Coomassie Blue and de-stained with 50% methanol:7% acetic acid. The same gels were then stained for glycoproteins with $^{125}$I-concanavalin A (Con A) (Robinson *et al.*, 1975). After being de-stained for Coomassie Blue the gels were equilibrated with 100mM Tris HCl, pH 7·5, 1mM $CaCl_2$ and 1mM $MnCl_2$. The equilibrated gels were then immersed in a bath containing 500 ml of the above buffer with 0·5 mg $^{125}$I-Con A (Pharmacia Fine Chemicals, Uppsala; $10^7$ ct/min) and 5 mg cytochrome C (Sigma, London). After 12 h at 4°C with gentle shaking, the gels were washed $\times 3$ with equilibration buffer, dried on to filter paper and autoradiographed directly. Glycoproteins were visible after $\sim$16 h autoradiography.

*Membrane isolation and solubilisation*

Two $\times 10^9$ sarcoma cells were prepared in suspension as described above from $\sim$30 g of *in vivo*-grown tumour. These cells were 100% viable as judged by Trypan-blue exclusion. After washing $\times 3$ in PBS the cells were suspended in 40 ml of a hypotonic buffer (10mM Tris HCl, pH 7·5, containing 0·1mM phenylmethyl sulphonyl fluoride (PMSF)) to inhibit endogenous protease activity. After standing on ice for 30 min, the cell suspension was given 20 strokes in a Dounce homogenizer and spun at 108,000 *g* for 1 h at 4°C in a swinging-bucket centrifuge on a layer of 45% sucrose. The membrane fraction layered on top of the sucrose and was collected, resuspended in 0·01M phosphate-buffered saline (PBS) and pelleted by centrifugation for 30 min at 108,000 *g*. The pellet was broken by pipetting with a Pasteur pipette and dissolved in 2 ml of 1% deoxycholate (DOC) in 0·01M PBS containing 0·1mM PMSF. After 2 h of gentle mixing at 4°C, 2 ml of 0·01M PBS was added, bringing the DOC concentration to 0·5%. This mixture was spun for 30 min at 108,000 *g* and the supernatant harvested.

*Removal of DOC*

The supernatant was dialysed in Visking tubing 8-1/32 at 4°C for 48 h with 3 changes in the dialysate (0·01M PBS and 0·1mM PMSF). This was effective in removing most of the DOC present, and made the preparations suitable for the biological assays. The effective removal of DOC was demonstrated by following the radioactivity released from the dialysis bag after the addition of a trace amount of $^{14}C$-labelled DOC (Radiochemical Centre, Amersham). The removal of DOC caused aggregation, as demonstrated by a cloudy precipitate. The whole preparation was used for immunization. The resultant membrane and deoxycholate-solubilized (DOCSOL) preparations were stored in aliquots at −20°C and used within one month of production. A summary of the production process appears in Fig. 1.

30 g Fresh tumour

↓

Trypsinize

↓

$2 \times 10^9$ Cells in suspension

↓

Resuspend in 40 ml hypotonic buffer with PMSF

0°C 30 min

↓

Homogenize

Spin 108,000 *g* 4°C 60 min on sucrose cushion

↓

Harvest membranes at interface

Wash in 0·01M phosphate saline (PBS)

↓

108,000 *g* 4°C 30 min to pellet

↓

Take up in 1% DOC in PBS

Agitate 2 h

↓

Add PBS to obtain 0·5% DOC

↓

Spin 108,000 *g*, 4°C 30 min

↓ ↓

Supernatant Pellet

↓

Dialyse 60 h against PBS + PMSF

↙ ↘

Assay Store at —20°C

FIG. 1.—Preparation and deoxycholate solubilization of tumour membranes.

*Lectin column preparation*

Concanavalin A (Con A), wheat germ agglutinin (WGA: Pharmacia Fine Chemicals, Uppsala, Sweden), *Ricinus communis* agglutinin (RCA: Miles Laboratories, Stoke Poges) and *Lens culinaris* haemagglutinin (LCH, prepared from lentils by the recipe of Howard *et al.*(22)) were coupled to cyanogen-bromide-activated Sepharose 4B (Pharmacia Fine Chemicals, Uppsala). Two g of Sepharose-4B cyanogen-activated gel was swollen and washed for 15 min in 500 ml 1mM HCl on a sintered glass filter. 10–50 mg of the lectin to be coupled was dissolved in 10 ml of the coupling buffer (0·1M $NaHCO_3$, 0·5M NaCl, pH 8·3), which included 2% of the specific sugar recognized by the lectin. The washed, swollen gel was added to the coupling buffer and mixed for 2 h at room temperature (19°C). Unbound material was washed away with coupling buffer and any remaining active groups were reacted with 1M ethanolamine (pH 8·0) for 1 h. Three washing cycles were used to remove non-covalently absorbed protein, each cycle consisting of a wash at pH 4·0 (0·1M sodium acetate, 1·0M NaCl) followed by a wash in coupling buffer. This procedure produced the binding of 70–80% of the lectin to the Sepharose as estimated from the optical density at 280 nm (OD 280) of added and unbound lectin.

*Running of columns*

Five ml of washed lectin-coupled gel was loaded into a plastic syringe on top of a small piece of glass wool. The column was equilibrated with loading buffer (0·01M $NaHPO_4$, $10^{-5}$M $CaCl_2$, $10^{-5}$M $MnCl_2$, 0·1mM PMSF, 0·85% NaCl, 0·2% DOC, pH 7·3) and run at 25°C. DOCSOL was loaded in a 2 ml volume and allowed to equilibrate for 30 min. The column was then washed with loading buffer until no further protein (determined by OD 280 of the effluent) was detected. Three washings, containing unbound components, were pooled. The eluting buffer, containing 2% of the sugar binding specifically to the lectin (α-methyl mannose for Con-A and LCH, N acetyl glucosamine for WGA and D-galactose for RCA) was then run into the column. After 30 min equilibration the column was washed with eluting buffer and again the washings collected and pooled (bound fraction).

Duplicate columns of Con A, LCH, RCA and WGA-coupled sepharose were prepared and used to remove glycoproteins from B10/MC6A and 6B DOCSOL respectively. Columns were loaded with 2 ml DOCSOL prepared from $10^9$ cells. Bound and unbound fractions were collected, dialysed for 48 h against 0·01M PBS and concentrated by vacuum dialysis. Aliquots (0·1M) were removed for estimation of OD 280, H-2$^b$, and for running on SDS–PAGE. The remainder was divided into 3 portions and stored at −20°C. Later groups of 3 B10 mice were immunized by 3 weekly s.c. injections of 0·2 ml of each stored fraction into the right groin and challenged 10 days after the last immunization with $10^5$ MC sarcoma cells.

### RESULTS

### *H-2 release*

The relative amount of H-2$^b$ released, as determined by inhibition of a B10BR anti-B10 (anti-H-2$^b$) lymphocytotoxic serum, is shown for MC6A cells in Fig. 2.

FIG. 2.—H-2$^b$ released from MC6A cells by membrane solubilization. The total amount of H-2$^b$ is expressed in relation to the absorption capacity of the membranes (100%). These absorptions were performed by using the amount of membranes or DOC-solubilized material produced from $10^7$ cells after dialysis to remove DOC. The pellet remaining after centrifugation was taken up in a further volume of 1% DOC in 0·01M PBS and spun again for 30 min at 108,000 *g*. Supernatant was removed and an absorption assay performed (DOC 2). The pellet was again taken up in 1% DOC and the process repeated twice (DOC 3 and 4).

For reasons not understood, preparation of membranes from cells apparently increased the amount of antigen available to absorb anti-H-2 activity. A single extraction of these membranes with DOC releases the H-2$^b$ absorption equivalent of that of intact cells. Further DOC extraction of the undissolved material solubilizes only small additional amounts of H-2. For this reason only one DOC extraction was performed. That MC 6A DOCSOL was unable to absorb an anti-H-2$^k$ serum indicates the specificity of DOC-released material.

### *Characterization of the solubilized membranes on acrylamide gels*

Fig. 3 shows a comparison of the proteins and glycoproteins (Con A-staining proteins) in the material during the various stages of solubilization. The majority of the proteins from the whole-cell extract are not in the membrane preparation. Furthermore, solubilization of the membranes results in the removal of several other major proteins from the preparation. In contrast the glycoprotein profiles of the membranes and the DOCSOL are very similar, showing that solubilization of glycoprotein is efficient. Comparison of the protein profiles of the preparations shows

FIG. 3.—Polyacrylamide-gel electrophoresis of MC6B cells (1), membranes (2) and DOCSOL (3). Staining by Coomassie blue or $^{125}$I Con-A.

FIG. 4.—Growth of MC6A and MC6B in mice immunized with various preparations. Groups of 4 mice were immunized by 3 weekly s.c. injections into the right groin of $10^7$ irradiated cells ▲; membranes ○, DOCSOL □, each containing the amount of H-$2^b$ equivalent to $4 \times 10^7$ cells. In a and b, MC6A preparations were the immunogen: in c and d, MC6B preparations. Ten days after the last immunization the animals were challenged with $2 \times 10^5$ viable MC6A or 6B cells in 0·2 ml PBS s.c. into the right groin. A group of unimmunized animals was challenged concurrently, ●. Bars represent s.e. (similar values for b and c omitted for clarity).

that the detergent extraction provides a product which is purified with respect to whole cells. Furthermore there is no evidence of extensive degradation of either proteins or glycoproteins.

*TSTA release*

Figs. 4 (a–d) show the growth curves of a challenge tumour, either MC6A or MC6B, after immunization of the recipient mice with either irradiated whole cells, membranes or solubilized membranes

FIG. 5.—Response to varying doses of irradiated cells or DOCSOL from B10 MC6A after 1 or 3 weekly immunizations.

of either MC6A or 6B origin. The dose–response curve for immunization with either cells or DOCSOL is shown in Fig. 5. It can be seen that membranes and the soluble material are immunogenic, although less so than whole cells. This may reflect loss during the extraction process or the weaker immunogenicity of the subcellular preparations. The specificity between MC6A and 6B is maintained in these preparations.

*Lectin column fractionation*

H-$2^b$ bound to both Con A and LCH columns, but not to those made from RCA or WGA Sepharose. Rejection assays performed after immunization of mice with various column fractions revealed TSTA in the bound fraction of the WGA column and in the unbound fractions on Con A, LCH and RCA columns. These results are presented as growth curves in Fig. 6, and as antigenic ratios in the Table. The binding of TSTA to WGA permits considerable purification in a single step. Furthermore, by using a combination of Con A and WGA columns, separation of TSTA from H-2 and proteins that can bind to both columns could be achieved. The individual specificities of TSTA for 6A or 6B tumours was conserved in the purified preparations. In a further experiment, the fraction of MC6A DOCSOL that did not bind to

FIG. 6.—Protection by bound and unbound fractions from Con A and WGA Sepharose columns. B10/MC6A or MC6B DOCSOL prepared from $10^9$ cells was fractionated on Con A and WGA Sepharose. Groups of 3 mice were immunized with 3 weekly injections of bound (△) or unbound fractions (○). Ten days after the last immunization the animals were challenged with $2 \times 10^5$ autologous tumour cells. A group of unimmunized animals (○) was challenged concurrently. (Bars indicate ±s.e.)

immobilized Con A (C⁻) was run on a WGA column. The fraction binding to the latter column ($C^-$ $W^+$) was then assayed for TSTA activity. The rejection assay results are shown in the Table. Again specificity is conserved. The $C^-$ $W^+$ fraction is clearly the most refined form of TSTA that we prepared.

### DISCUSSION

The characterization of the diverse set of tumour-specific transplantation antigens (TSTA) of chemically induced sarcomas has been hindered by the lack of methods for preparing them in a form suitable for comparison with each other and with other cell-surface proteins. This requires releasing them in good yield from the tumour cells in a form that is undegraded and retains the specificity they show on intact cells.

TABLE.—*Immunogenicity of lectin-column fractionated DOCSOL of MC6A and MC6B sarcomas*

| Fraction | | Antigenic ratio on challenge with MC6A | MC6B |
|---|---|---|---|
| Con A bound | MC6A | 1·1 | 0·9 |
| Con A unbound | | **2·3** | 1·1 |
| Con A bound | MC6B | 1·2 | 1·0 |
| Con A unbound | | 1·0 | **1·9** |
| LCH bound | MC6A | 1·0 | 0·9 |
| LCH unbound | | **3·2** | 1·3 |
| LCH bound | MC6B | N.T. | 0·9 |
| LCH unbound | | N.T. | **1·8** |
| RCA bound | MC6A | 1·0 | N.T. |
| RCA unbound | | **1·8** | N.T. |
| RCA bound | MC6B | N.T. | 1·0 |
| RCA unbound | | N.T. | **1·9** |
| WGA bound | MC6A | **1·9** | 1·0 |
| WGA unbound | | 1·0 | 0·9 |
| WGA bound | MC6B | 1·1 | **1·9** |
| WGA unbound | | 1·2 | 0·9 |
| $C^-W^-$ | MC6A | 1·0 | N.T. |
| $C^-W^+$ | | **3·1** | 1·2 |

We have shown that the use of a mild detergent in the presence of proteolysis inhibitor does satisfy the desired criteria. The material released remains antigenic in transplantation assays and shows the same specificity as the original tumour. Whilst it is easy to show serologically that H-2 is released in very high yield, it is not possible to make such quantitative statements about TSTA. We lack antisera specific for TSTA, though recent experiments on MC murine sarcomas (Parker & Rosenberg, 1977; De Leo *et al.*, 1977) show that these might be possible to prepare. Lacking this, the only assay we used for TSTA was that of immunizing mice against tumour challenge. This assay does not lend itself to the quantitation of TSTA yielded in the various purification steps. Nonetheless, the PAGE analyses seem to indicate that the solubilization process does not lead to loss or degradation of any of the major membrane proteins.

The binding of TSTA to WGA columns provides a relatively simple procedure for considerable purification for the 2 tumours studied. It will be interesting to see whether this feature will hold for other tumours. In view of the question whether TSTAs are similar molecules to H-2, their evident separations on WGA and Con A columns is of interest. While this allows us to say nothing about the protein moieties of these molecules, it does at least indicate something about their exposed sugars. We hope to continue this comparison in further experiments.

We thank Philip Wright, Pam Gregory, Sue Ingle and Lorraine Carver for expert assistance. K.S. held an MRC Training Fellowship.

### REFERENCES

BASOMBRIO, M. A. (1970) Search for common antigenicities among twenty-five sarcomas induced by methylcholanthrene. *Cancer Res.*, **30**, 2458.

BRIDGEN, J., SNARY, D., CRUMPTON, M. J., BARNSTABLE, C., GOODFELLOW, P. & BODMER, W. F. (1976) Isolation and N terminal amino acid sequence of membrane bound HLA antigens. *Nature*, **261**, 200.

DAVID, G. S. & REISFELD, R. A. (1974) Binding of carcinoembryonic antigen to concanavalin A-sepharose. *J. Natl Cancer Inst.*, **53**, 1005.

DE LEO, A. B., SHIKU, H., TAKAHASHI, T., JOHN, M. & OLD, L. J. (1977) Cell surface antigens of chemically induced sarcomas of the mouse. *J. Exp. Med.*, **146**, 720.

GURD, J. W. & MAHLER, H. R. (1974) Fractionation of synaptic plasma membrane glycoproteins by lectin affinity chromatography. *Biochemistry*, **13**, 5193.

HENRIKSEN, O., ROBINSON, E. A. & APPELLA, E. (1978) Structural characterisation of H-2 antigens purified from mouse lines. *Proc. Natl Acad. Sci.*, **75**, 3322.

HOLMES, E. C., MORTON, D. L., SCHIDLOVSKY, G. & TRAHAN, E. (1971) Cross reacting tumor specific transplantation antigens in methylcholanthrene induced guinea pig sarcomas. *J. Natl Cancer Inst.*, **46**, 693.

LAEMMLI, U. K. (1970) Cleavage of structural proteins during the assembly of the head of bacteriophage T4. *Nature*, **227**, 680.

LAW, L. W., APPELLA, E. & HENRIKSEN, O. (1976) Some biologic and biochemical properties of soluble tumor antigens. *Ann. N.Y. Acad. Sci.*, **276**, 11.

LOTAN, R., BREATHIE, G., HUBBELL, W. & NICOLSON, G. L. (1977) Activities of lectins and their immobilized derivatives in detergent solutions. *Biochemistry*, **16**, 1787.

MCCOLLESTER, D. L. (1970) Isolation of Meth A cell surface membranes possessing tumor-specific transplantation antigen specificity. *Cancer Res.*, **30**, 2832.

MELTZER, M. S., OPPENHEIM, J. J., LITTMAN, B. H., LEONARD, E. J. & RAPP, H. J. (1972) Cell mediated tumor immunity measured *in vitro* and *in vivo* with soluble tumor specific antigens. *J. Natl Cancer Inst.*, **49**, 727.

NATORI, T., LAW, L. W., APPELLA, E. (1977) Immunochemical evidence of a tumor specific surface antigen obtained by detergent solubilisation of the membranes of a chemically induced sarcoma, MetnA. *Cancer Res.*, **37**, 3406.

OETTGEN, H. F., OLD, L. J., MCLEAN, E. P. & CALDWELL, E. A. (1968) Delayed hypersensitivity and transplantation immunity elicited by soluble antigens of chemically induced tumors in inbred guinea pigs. *Nature*, **220**, 295.

PARKER, G. A. & ROSENBERG, S. A. (1977) Serologic identification of multiple tumor associated antigens on murine sarcomas. *J. Natl Cancer Inst.*, **58**, 1303.

PELLIS, N. R. & KAHAN, B. D. (1975) Specific tumor immunity induced with soluble materials: Restricted range of antigen dose and of challenge tumor load for immuno-protection. *J. Immunol.*, **115**, 1717.

ROBINSON, P. J., BULL, F. G., ANDERTON, B. H. & ROITT, I. M. (1975) Direct autoradiographic visualisation in SDS gels of lectin binding components of the human erythrocyte membrane. *FEBS Letters*, **58**, 330.

SEIGERT, W., FENYO, E. M. & KLEIN, G. (1977) Separation of the Moloney virus determined cell surface antigen (MCSA) from known virion proteins associated with the cell membrane. *Int. J. Cancer*, **20**, 75.

SHARON, N. & LIS, H. (1972) Lectins: cell agglutinating and sugar specific proteins. *Science*, **177**, 949.